# A VIRTUAL SUPPORT GROUP FOR PERSONS LIVING ALONE WITH ALZHEIMER’S DISEASE

**DOI:** 10.1093/geroni/igz038.1431

**Published:** 2019-11-08

**Authors:** Allison Gibson

**Affiliations:** University of Kentucky, Lexington, Kentucky, United States

## Abstract

To date, early stage programming for the persons with the disease has been limited. The purpose of this study was to explore the challenges and opportunities of a virtual support group intervention for persons living alone with Alzheimer’s disease (AD). Following recruitment, participants who were newly diagnosed (within 2 years of diagnosis) and residing alone in their own residence were included in a pilot study of a virtual support group intervention for a 3-month period. Data were collected before and after the intervention through the use of surveys and one-on-one interviews with all 12 participants (n=12). Data were analyzed using a mixed methods approach including thematic analysis. Results indicated that the virtual support group intervention increased group members’ education of the disease, knowledge regarding care planning, feelings of empowerment towards the diagnosis of the disease, and increased feelings of social support. Implications for such interventions will also be discussed.

